# Determinants of Oral Health System Effectiveness for Preschool Children in Decentralized Child Development Centres: A Cross-Sectional Study in Northeastern Thailand

**DOI:** 10.3390/epidemiologia7040098

**Published:** 2026-07-11

**Authors:** Somporn Keawtong, Chanwit Maneenin, Adisorn Wongkongdech, Niruwan Turnbull

**Affiliations:** 1 Sirindhorn College of Public Health, Ubon Ratchathani 34190, Thailand; somporn.kea@msu.ac.th; 2 College of Medicine and Public Health, Ubon Ratchathani University, Ubon Ratchathani 34190, Thailand; chanwit.m@ubu.ac.th; 3 Faculty of Public Health, Mahasarakham University, Maha Sarakham 44000, Thailand; adisorn.w@msu.ac.th

**Keywords:** oral health systems, early childhood caries, preschool children, health system determinants, Thailand

## Abstract

Background: Early childhood oral health remains a major public health concern in Thailand despite the implementation of preventive oral health programs. Understanding the factors influencing oral health care system effectiveness is essential for improving oral health outcomes among preschool children. This study aimed to assess oral health care system factors and identify determinants associated with the effectiveness of oral health care services for preschool children in child development centres under local administrative organizations in Ubon Ratchathani Province, Thailand. Methods: A cross-sectional analytical study was conducted among 270 stakeholders involved in the preschool oral health care system, including administrators, teachers, health professionals, village health volunteers, and parent representatives. Data were collected using a structured questionnaire developed based on the Input–Process–Output framework. Oral health outcome data were obtained from routine dental examination records of 498 preschool children. Descriptive statistics, Pearson’s correlation analysis, and multiple linear regression were used to analyse the data. Results: Overall oral health care system effectiveness was rated at a high level (mean = 4.01, SD = 0.44). Service delivery processes showed the highest mean score (mean = 4.19, SD = 0.39), while monitoring and evaluation activities demonstrated the lowest performance. The prevalence of dental caries among preschool children was 57.83%. Multiple linear regression analysis identified service delivery processes (β = 0.239, *p* < 0.001) and home visits by public health and dental personnel (β = 0.165, *p* = 0.006) as significant predictors of oral health care system effectiveness. The final model explained 11.1% of the variance in system effectiveness (R^2^ = 0.111, *p* < 0.001). Conclusions: The effectiveness of oral health care systems for preschool children was primarily influenced by the quality of service delivery processes and community-based outreach activities. These findings indicate that strengthening service implementation and home-visit programs may enhance oral health system effectiveness in child development centres operating within decentralized local government settings.

## 1. Introduction

Early childhood caries (ECC) is the most prevalent chronic disease among preschool-aged children globally and represents a significant public health challenge with far-reaching consequences beyond the oral cavity [1]. The World Health Organization estimates that the global prevalence of dental caries among children aged 1–9 years is approximately 42.71%, increasing to 44.61% in lower-middle-income countries, reflecting persistent inequities in oral health outcomes across socioeconomic contexts [2]. ECC is now widely recognized as a multifactorial condition influenced by biological, behavioural, socioeconomic, and environmental determinants, with long-term impacts on nutritional status, physical development, cognitive performance, and overall quality of life [2,3,4]. Furthermore, early caries experience is a strong predictor of future oral health trajectories and health-seeking behaviours across the life course, underscoring the importance of early prevention and system-level intervention [3].

In Thailand, the burden of ECC remains critically high despite ongoing national policy efforts. Data from the Ministry of Public Health Thailand indicate that the prevalence of dental caries among 5-year-old children is 75.6%, with a mean decayed, missing, and filled teeth (dmft) index of 4.5, exceeding global averages and highlighting Thailand as a high-burden setting [3]. Regional disparities persist, with higher prevalence observed in northern and southern regions compared to other parts of the country [5]. In response, Thailand has implemented the 4D oral health policy framework—Development, Diet, Dental, and Disease, which emphasizes integrated, multi-sectoral approaches to early childhood oral health promotion [6]. Since 2016, administrative responsibility for child development centres (CDCs) has been decentralized to local administrative organizations (LAOs), positioning these entities as key actors in planning, financing, and delivering oral health services for preschool children [7].

Existing literature highlights several critical determinants influencing oral health outcomes in preschool populations. Structured interventions such as supervised tooth brushing programs in institutional settings have demonstrated significant reductions in dental plaque and caries incidence [8]. Caregiver knowledge, attitudes, and practices are also consistently identified as major determinants of children’s oral health behaviours, with evidence showing that parents possessing adequate knowledge and positive attitudes are significantly more likely to adopt appropriate oral health practices for their children (ORadj = 4.28; 95% CI: 1.61–11.40) [9,10]. However, a substantial gap persists between need and utilization of dental services. For instance, one study reported that although 76.8% of children required urgent dental care, 98.5% of caregivers had never taken their child to a dental facility, indicating significant barriers to access and continuity of care [9]. Studies conducted in Thai CDCs further report high caries prevalence ranging from 58.11% to 80.26%, confirming that institutional settings remain high-risk environments for ECC despite the presence of formal preventive programs [11,12].

International evidence suggests that oral health outcomes among preschool children are strongly influenced by the organization and performance of health systems. In Australia, community-based oral health promotion programs integrating parental education, fluoride varnish application, and early risk assessment have demonstrated significant improvements in preventive service uptake and reductions in untreated dental caries among disadvantaged populations. Similarly, the Child smile programme in Scotland has shown that combining supervised tooth brushing, community outreach, and preventive dental services can substantially reduce socioeconomic inequalities in oral health outcomes among young children [13,14]. In Brazil, the integration of oral health teams into primary healthcare through the National Oral Health Policy has improved access to preventive services; however, challenges remain regarding continuity of care and regional disparities in service provision [15,16]. These international experiences highlight the importance of examining oral health systems not only from a clinical perspective but also through the broader lens of governance, service delivery, and community engagement.

Despite the growing body of evidence on individual and behavioural determinants, there remains a critical gap in understanding system-level factors influencing oral health care effectiveness. Most studies in Thailand and other low- and middle-income countries have focused primarily on clinical outcomes and individual behaviours, with limited attention to integrated health system components such as resource inputs, service delivery processes, system outputs, and environmental context [12,17,18]. This gap is particularly important in decentralized health systems, where variability in governance, resource allocation, and implementation capacity across LAOs may significantly influence service effectiveness. The northeastern region of Thailand, characterized by socioeconomic constraints and disparities in health infrastructure, provides a particularly relevant context for examining these system-level dynamics [17,18].

Ubon Ratchathani Province, the largest province in northeastern Thailand, offers a diverse and representative setting encompassing urban, peri-urban, and rural administrative structures. The province includes a wide range of LAO types and over 800 CDCs, making it an ideal context for examining oral health system performance under decentralized governance. Therefore, this study applies an Input–Process–Output (IPO) health systems framework to investigate factors affecting oral health care system effectiveness for preschool children in CDCs under LAOs. The study aims to (1) assess the current status of the oral health care system, including strengths and weaknesses across system components, and (2) identify key determinants associated with system effectiveness using multivariate statistical analysis. By addressing this critical evidence gap, the findings are expected to inform policy development, resource allocation, and program design for improving oral health outcomes among preschool children in decentralized health systems.

## 2. Materials and Methods

### 2.1. Study Design and Setting

A cross-sectional analytical study was conducted across child development centres (CDCs) under local administrative organizations (LAOs) in Ubon Ratchathani Province, northeastern Thailand, between November 2023 and January 2024. The province was selected as the study setting because it is the largest province in the northeastern region and encompasses a heterogeneous distribution of LAO types including municipalities (Thesaban) and sub-district administrative organizations (SAOs) operating across urban, peri-urban, and rural areas, providing both ecological validity and policy relevance.

### 2.2. Study Population and Sampling

The target population comprised all stakeholders directly involved in the preschool oral health care system within child development centres (CDCs) operating under local administrative organizations (LAOs) in Ubon Ratchathani Province, Thailand. To ensure comprehensive system-level representation, six stakeholder groups were defined a priori based on their functional roles within the oral health care system: (1) LAO chief administrators and education division directors, responsible for governance, policy implementation, and budget allocation; (2) CDC committee members, providing institutional oversight and advisory functions; (3) public health personnel and dental staff, delivering clinical and preventive oral health services; (4) childcare teachers, implementing daily oral health promotion activities; (5) parent representatives, reflecting household-level caregiving practices and health behaviours; and (6) village health volunteers (VHVs), serving as community-based health promotion agents linking households and the health system.

The required sample size was determined using Cohen’s formula [17] for multiple regression analysis, which is appropriate for studies examining the influence of multiple predictors on a continuous outcome. Based on an anticipated medium effect size (R^2^ = 0.15), eight independent variables, a significance level (α) of 0.05, and a statistical power of 0.80, the minimum sample size was calculated to be 233 participants. To enhance statistical reliability and compensate for potential non-response and incomplete data (estimated at approximately 15%), the final sample size was increased to 270 participants.

A four-stage random sampling technique was employed to ensure representativeness across geographic and administrative contexts. In Stage 1, all 25 districts in Ubon Ratchathani Province were stratified into three area types—urban, peri-urban, and rural—based on the Department of Local Administration classification system. One district from each stratum was then selected using simple random sampling, resulting in the inclusion of Warin Chamrap District (urban), Muang Sam Sip District (peri-urban), and Na Yia District (rural). In Stage 2, LAOs within each selected district were sampled using proportional stratified random sampling according to LAO type (municipalities and sub-district administrative organizations). In Stage 3, CDCs within each selected LAO were selected through simple random sampling, proportionate to the total number of centres in each area type to maintain distributional balance, and in Stage 4, the total sample size (*n* = 270) was proportionally allocated across the six stakeholder groups based on their relative representation within the system. Stakeholder groups with a finite population within selected units, namely LAO administrators, CDC committee members, public health personnel, dental staff, and childcare teachers, were recruited using purposive sampling by including all eligible individuals. In contrast, parent representatives and VHVs, who constitute larger and more variable populations, were selected using simple random sampling from official lists provided by each CDC. This combined sampling approach ensured both structural representativeness of the oral health care system and adequate statistical power for multivariate analysis.

### 2.3. Outcome Variable and Independent Variables

The primary outcome variable was oral health care system effectiveness (SUMO), defined as the composite score reflecting the degree to which the CDC oral health care system achieves its intended service outputs, operationalized across three dimensions: (1) proportion of children receiving annual oral health examinations; (2) proportion receiving preventive interventions (fluoride varnish); and (3) completeness of treatment follow-up and disease surveillance. Higher SUMO scores indicate greater system effectiveness.

Eight independent variables were examined, organized within the Input–Process–Output (IPO) systems framework [18] and an adapted social determinants of health model [18]: input factors (SUMI: personnel, budget, and equipment readiness), service delivery process factors (SUMP: comprehensiveness and systematicity of in-centre oral health activities), environmental factors (SUME: organizational and physical environment within CDCs), dental health media support (HE: receipt of oral health educational materials and equipment from external agencies), dental service accessibility (WHS: access to and utilization of external dental services), mode of transport to dental facilities (THS), home visits by public health and dental personnel (CHS), and dental health equipment support from external sources (FOHS).

### 2.4. Research Instrument

The questionnaire was developed based on the Input–Process–Output (IPO) framework, which conceptualizes health system performance as a function of available resources (inputs), service implementation activities (processes), service achievements (outputs), and contextual environmental factors. The framework was adapted to reflect the organizational structure and operational characteristics of oral health services delivered through child development centres under decentralized local government administration [19]. A structured questionnaire was developed by the research team, grounded in the IPO systems framework and a systematic review of relevant literature. The instrument comprised three sections. Section 1 collected personal and contextual demographic data through seven items (sex, age, marital status, educational attainment, occupation, monthly income, and stakeholder role). Section 2 assessed oral health care system factors using 18 binary checklist items (1 = present, 0 = absent), organized across input, process, and output components. Item-level scores were aggregated and interpreted using the criterion-referenced classification developed by Bloom et al. [20]: low (<60%), moderate (60–79%), and high (≥80%), and Section 3 assessed social, economic, and environmental access factors through 11 items covering dental service accessibility, agency support, health education material provision, and the organizational environment of the CDC.

Instrument quality was established through two sequential procedures. Content validity was evaluated by a panel of five domain experts in dental public health, health systems research, and educational measurement, using the Item-Objective Congruence (IOC) index [21]. All items attained IOC values of ≥0.50, the accepted minimum threshold for content validity. Internal consistency reliability was assessed through a pilot study with 30 participants from CDCs in Detudom District, Ubon Ratchathani Province, a site geographically separated from the main study areas and administratively excluded from the sampling frame [22]. Cronbach’s alpha coefficients were 0.72 for the health service system section, 0.77 for the socioeconomic section, and 0.71 for the environmental section, all meeting or exceeding the conventional threshold of 0.70 [23].

### 2.5. Data Collection Procedures

Data collection was conducted between November 2023 and January 2024 by the principal investigator (S.K.) and four trained research assistants. The study utilized data from two complementary participant groups. The first group comprised 270 stakeholders directly involved in the preschool oral health care system, including local administrative organization administrators, education division directors, child development centre committee members, childcare teachers, public health personnel, dental personnel, village health volunteers, and parents or caregivers. Data from this group were collected using structured questionnaires to assess oral health care system factors and determinants of system effectiveness. The second group comprised 498 preschool children enrolled in the participating child development centres. Data from this group were obtained from routine annual oral health examination records maintained by child development centres and local health facilities. These records were used to assess oral health outcomes, particularly dental caries prevalence among preschool children.

Prior to fieldwork, all research assistants participated in a structured training session covering study procedures, participant communication, questionnaire administration, and quality assurance protocols to ensure consistency throughout the data collection process. Oral examinations of preschool children were conducted by licensed dental personnel, including dentists and dental nurses, during routine outreach oral health service visits at the child development centres. Dental caries in primary dentition was assessed according to the World Health Organization (WHO) diagnostic criteria, and caries experience was recorded using the decayed (d), missing (m), and filled (f) teeth (t) (dmft) index. Dental caries prevalence was calculated as the proportion of examined children presenting with at least one decayed, missing due to caries, or filled primary tooth. Examinations were performed under standardized field conditions using portable dental examination equipment and adequate illumination in accordance with national oral health surveillance guidelines.

Formal written permission was obtained from each selected local administrative organization and district health office before data collection commenced. Eligible stakeholders received written and verbal explanations regarding the study objectives, procedures, confidentiality, voluntary participation, and the right to withdraw without consequence. Written informed consent was obtained from all participants before questionnaire administration. Questionnaires were self-administered and completed questionnaires were checked for completeness on the day of collection.

### 2.6. AI-Assisted Language Editing

AI-assisted language editing was used to improve grammar, clarity, and readability during manuscript preparation. All scientific content, study design, data collection, statistical analyses, interpretation of the results, and final wording were critically reviewed, verified, and approved by the authors, who take full responsibility for the content of this manuscript.

### 2.7. Statistical Analysis

All statistical analyses were performed using IBM SPSS Statistics, Version 26.0 (IBM Corp., Armonk, NY, USA). Analysis proceeded in two stages. In the first stage, descriptive statistics including frequencies, percentages, means, and standard deviations were used to characterize the study sample and to profile each oral health care system factor across input, process, output, and environmental dimensions.

In the second stage, inferential analyses were applied to examine associations and predictive relationships among variables, with the significance threshold set at *p* < 0.05 (two-tailed). Pearson’s product-moment correlation coefficients were computed to assess the direction and magnitude of bivariate associations between each independent variable and SUMO, and to screen for potential multicollinearity among predictors. Predictors with inter-correlations exceeding r = 0.70 were flagged for further examination. Multiple linear regression analysis using the simultaneous Enter method in which all eight predictor variables were entered in a single block was subsequently conducted to identify statistically significant independent predictors of SUMO [22]. Model fit was evaluated using the coefficient of determination (R^2^) and adjusted R^2^. Variance Inflation Factor (VIF) values were inspected for all predictors; VIF values < 5.0 were considered indicative of acceptable multicollinearity.

### 2.8. Ethical Approval

This study was reviewed and approved by the Human Research Ethics Committee of Mahasarakham University prior to data collection (Approval No. 423-410/2023). All procedures were conducted in full accordance with the ethical principles of the Declaration of Helsinki (2013 revision). Written informed consent was obtained from all participants before enrolment. Data were anonymized at the point of collection, stored on password-protected institutional servers, and used exclusively for academic and research purposes. Parents and caregivers of enrolled children were explicitly informed that participation was voluntary and bore no bearing on the services provided to their children.

## 3. Results

### 3.1. Participant Characteristics

A total of 270 stakeholders participated. The sample was predominantly female (*n* = 227, 84.07%), with a mean age of 42.26 years (SD = 6.76; range 28–60 years). The largest age group was 41–50 years (43.33%), followed by 31–40 years (41.48%), over 50 years (12.59%), and below 31 years (2.59%). Most participants were married or cohabiting (65.93%).

Regarding educational attainment, most held a bachelor’s degree or equivalent (37.78%), followed by a vocational diploma at the associate level (25.93%). Monthly income for the majority fell within 5000–17,490 Thai Baht (69.63%), with a median of 10,000 Baht. Stakeholder roles included CDC committee members, VHVs, and parent representatives (22.22% each, *n* = 60 per group), childcare teachers (11.11%), dental personnel (10.37%), education division directors (7.41%), LAO chief administrators (3.71%), and public health officers (0.74%). (See Table 1).

### 3.2. Oral Health Care System Factors

#### 3.2.1. Input Factors

Input factors covering personnel, budget, and equipment readiness demonstrated strong performance in human resource and equipment domains, but critical weaknesses in financial resource allocation. Designated dental personnel were present in 99.63% of centres, capacity-building training had been provided in all centres (100%), adequate staffing for service delivery was reported in 97.04%, and a formally appointed oral health officer within the LAO was present in 90.37%. Equipment for oral health screening was available in 99.63% of centres, sufficient preventive supplies for all enrolled children were maintained in 94.07%, and age-appropriate educational materials for preschool children were available in 78.52% of centres.

In contrast, financial input factors represented the most critical deficiency at this level. Only 60.37% of centres reported budget support from the local health security fund for oral health promotion activities, and only 59.26% reported a sufficient budget for dental health material procurement both classified as moderate-level adequacy under Bloom’s criterion-referenced framework [20]. These findings indicate that human capital and physical equipment are relatively well-resourced, whereas dedicated financial resources for oral health remain the primary structural bottleneck in the input component of the system.

#### 3.2.2. Service Delivery Process Factors

Service delivery process factors exhibited a sharply polarized profile. Notable strengths were demonstrated across the majority of indicators: daily supervised tooth brushing after lunch (100%), cariogenic food and beverage control (96.67%), coordination with local dental personnel for service planning (94.44%), active promotion of parental engagement (94.81%), capacity-building activities for teaching and care staff (86.67%), oral health risk screening at child enrolment (90.00%), emergency response protocols for acute dental problems during school hours (83.70%), and referral systems for children with oral health problems (80.00%).

In marked contrast, systematic monitoring and evaluation of oral health activities was conducted in only 8.15% of centres, the single lowest-performing indicator across the entire system assessment, classified as a low-level deficiency under the criterion-referenced framework. This finding indicates a near-complete absence of data-driven quality assurance mechanisms at the process level. Without systematic evaluation infrastructure, the effectiveness of otherwise well-established activities cannot be reliably measured or continuously improved.

#### 3.2.3. Service Output Factors

Service output factors revealed a divergent pattern between preventive coverage and treatment continuity. Universal preventive coverage was achieved: all enrolled children (100%) received at least one oral health examination and fluoride varnish application per year, both indicators meeting the preventive coverage benchmarks recommended by the American Academy of Pediatric Dentistry [15]. Annual oral disease situation reports were produced by 96.30% of centres. Based on oral health examination records of preschool children enrolled in the participating child development centres, the prevalence of dental caries was 57.83%. Dental status was assessed using the dmft index during routine examinations conducted by dental personnel as part of annual oral health surveillance activities. This prevalence indicates that more than half of preschool children continued to experience dental caries despite universal coverage of preventive oral health services.

However, the treatment pathway showed substantial gaps. Referral of children with identified oral health problems to appropriate facilities occurred in only 58.89% of cases, and continuous disease prevention and treatment follow-up was maintained in fewer than half of centres (48.89%), both classified as low-level performance. These findings indicate that while the system performs well in early detection and primary prevention, it fails to ensure adequate care continuity for children with identified needs. Complete oral health care system factor data across all three components are presented in Table 2.

#### 3.2.4. Social, Economic, and Environmental Factors

##### Dental Service Access and Agency Support

With respect to dental service access, parents or caregivers were primarily responsible for accompanying children to dental appointments in 75.56% of cases (*n* = 204), while childcare teachers assumed this role in 24.44% (*n* = 66). The predominant mode of transport was motorcycle (53.70%), followed by private car (19.63%), hired vehicle (18.15%), and bicycle (8.52%).

Regarding agency support, 87.04% of CDCs reported having received oral health promotion supervision from public health or government personnel, and 94.07% had received dental health equipment and educational material support from relevant agencies in the past year. All CDCs (100%) received oral hygiene supplies; 96.67% received fluoride varnish application services; 96.30% received oral health examination services; and 95.56% received training sessions for parents and caregivers. Proportions receiving other forms of support were lower: oral health activity organization (64.81%), referral system support (65.19%), appointment and follow-up systems (62.59%), appropriate snack and meal arrangement (71.85%), and dental health handbooks for parents and teachers (35.56%). Pit and fissure sealant services were provided to 43.70% of centres, and LAO budget support to 51.48%.

The primary source of support was sub-district health promoting hospitals (81.48%), followed by LAOs (60.37%), community or general hospitals (37.41%), educational institutions (33.33%), district public health offices (28.89%), and provincial public health offices (28.52%). NGOs or foundations provided support to only 4.44% of centres. Despite high support receipt rates, only 9.63% of respondents rated the adequacy as more than sufficient, while 53.33% rated it as sufficient, 18.15% as partially sufficient, and 18.19% as insufficient. All CDCs (100%) had received at least one outreach oral health examination visit from a government health facility in the preceding year. Full data are presented in Table 3.

##### CDC Organizational Environment

Analysis of the CDC organizational environment revealed a generally favourable structural context for oral health care. Factors present in 80% or more of centres classified as high-level readiness included: oral health information and guidance provision to parents (97.04%), parental participation in the oral health care system (97.41%), regular joint meetings between CDC staff and dental personnel (96.30%), referral systems for children with oral health problems (95.19%), training plans for teachers and caregivers (94.07%), emergency protocols for acute dental problems during school hours (93.33%), individual oral health record-keeping (92.96%), oral health monitoring and follow-up systems (90.37%), integration of oral health content into the teaching curriculum (90.37%), directory of dental personnel and relevant agencies (90.00%), designated primary oral health officer (87.41%), dental equipment management systems (87.41%), written oral health policies or plans (85.93%), scheduled annual oral health promotion activity plans (84.81%), oral health risk screening at enrolment (84.44%), coordination systems with local dental health agencies (82.96%), annual budget plans for dental health supplies (81.11%), and scheduled outreach visit timetables (81.48%).

Areas requiring systematic improvement were concentrated in health information management and quality assurance mechanisms. The automated parental notification system for detected oral health problems was the most critically underdeveloped factor (22.22%), followed by the electronic dental health record management system (54.81%), the supervision system for monitoring staff oral health practice (58.52%), annual evaluation of oral health care system success (67.04%), systematic oral disease surveillance measures (72.22%), and regular assessment of children’s oral health knowledge and behaviours (74.44%). These moderate-to-low-performing factors share a common characteristic: they represent systematic feedback and accountability mechanisms whose absence undermines the capacity for data-driven quality improvement. Full data are presented in Table 4.

### 3.3. Correlation Analysis

Pearson’s product-moment correlation coefficients were computed among all independent variables and the outcome variable (SUMO) prior to multiple regression modelling. Three independent variables showed statistically significant positive correlations with SUMO: service delivery process factors (SUMP: r = 0.258, *p* < 0.01), input factors (SUMI: r = 0.186, *p* < 0.01), and environmental factors (SUME: r = 0.129, *p* < 0.05). The remaining five predictors dental health media support (HE), dental service accessibility (WHS), mode of transport (THS), home visits by public health personnel (CHS), and dental health equipment support (FOHS)—did not achieve statistical significance in bivariate association with SUMO.

Examination of inter-predictor correlations revealed several high associations warranting attention in the regression model. The strongest was observed between SUMP and CHS (r = 0.750, *p* < 0.01), indicating substantial shared variance between in-centre service delivery quality and home visit intensity. Strong associations were also found between CHS and FOHS (r = 0.650, *p* < 0.01) and between HE and WHS (r = 0.367, *p* < 0.01). These patterns were subsequently assessed via VIF diagnostics in the regression model. The complete correlation matrix is presented in Table 5.

### 3.4. Multiple Regression Analysis

Multiple linear regression analysis was performed using the Enter method to identify factors associated with oral health care system effectiveness. The overall regression model was statistically significant (F(6, 263) = 5.44, *p* < 0.001), with a multiple correlation coefficient (R) of 0.332. The model explained 11.1% of the variance in oral health care system effectiveness (R^2^ = 0.111; Adjusted R^2^ = 0.091), indicating that the included predictors had a modest but statistically significant contribution to system effectiveness.

Among the six predictor variables entered into the model, service delivery processes (SUMP) and home visits by public health and dental personnel (CHS) were identified as significant independent predictors of oral health care system effectiveness. Service delivery processes demonstrated the strongest positive association with system effectiveness (β = 0.458, *B* = 0.249, SE = 0.055, *t* = 4.523, *p* < 0.001), indicating that improvements in preventive service delivery, coordination of oral health activities, and routine implementation of oral health programs were associated with higher levels of overall system effectiveness.

Home visits by public health and dental personnel also showed a significant positive association with system effectiveness (β = 0.303, *B* = 0.725, SE = 0.270, *t* = 2.689, *p* = 0.008), suggesting that community-based outreach activities contribute to strengthening oral health care services for preschool children.

In contrast, input factors (SUMI), environmental factors (SUME), dental health media support (HE), and dental service accessibility (WHS) were not significantly associated with oral health care system effectiveness after adjustment for the other variables included in the model (*p* > 0.05).

Collinearity diagnostics indicated no evidence of problematic multicollinearity among the independent variables, with variance inflation factor (VIF) values ranging from 1.64 to 3.73, well below the commonly accepted threshold of 5.0. The detailed regression coefficients are presented in Table 6.

## 4. Discussion

This study provides a comprehensive systems-level analysis of factors influencing oral health care system effectiveness for preschool children in child development centres (CDCs) under local administrative organizations (LAOs) in northeastern Thailand. By applying an Input–Process–Output (IPO) framework, the findings highlight that system effectiveness is shaped less by the mere presence of resources and more by the quality of service delivery processes and the integration of community-based outreach mechanisms.

At the input level, the study identified strong performance in human resource readiness and equipment availability, with nearly universal presence of designated dental personnel and training provision. These findings are consistent with national policy directives and prior research emphasizing the importance of trained personnel and adequate infrastructure in supporting oral health promotion programs [6,10]. At the input level, the system showed strong human-resource and equipment readiness, with near-universal designated dental personnel (99.63%) and training provision (100%). In contrast, financial inputs were the weakest input element: only 60.37% of centres reported adequate budget from the local health security fund and 59.26% reported sufficient funds for material procurement, both within the moderate band of the criterion-referenced classification. Notably, however, input factors—including budget adequacy—were not statistically significant predictors of system effectiveness (β = 0.119, *p* = 0.114). Read together, the descriptive and regression results indicate that financial adequacy functioned as a relatively uniform necessary condition across centres rather than a factor that discriminated between more and less effective systems. The financing shortfall therefore represents a latent structural vulnerability that may not yet constrain measured outputs—because core preventive activities remain externally supported—but that threatens system sustainability should external support contract. This interpretation is consistent with evidence from Brazil’s decentralized oral health system, where local organizational and financing characteristics shaped the durability and continuity of child dental care rather than its immediate coverage [23].

In terms of service delivery processes, the study revealed a dual pattern of strengths and critical deficiencies. High adherence to preventive practices, such as daily supervised tooth brushing, dietary control, and coordination with dental personnel, reflects successful implementation of evidence-based interventions. These findings are consistent with previous studies demonstrating that structured tooth brushing programs can significantly reduce dental plaque and caries incidence among preschool children [8,13]. Moreover, strong parental engagement observed in this study reinforces existing literature identifying caregiver involvement as a key determinant of children’s oral health behaviours [9,10]. The importance of service delivery processes identified in this study is consistent with findings from several international health systems. In Scotland, the Childsmile programme demonstrated that systematic implementation of supervised tooth brushing, parental engagement, and preventive services within educational settings contributed to significant reductions in dental caries prevalence and oral health inequalities [11,12]. Similarly, evaluations of preschool oral health programs in Australia have highlighted that effective coordination between educational institutions, healthcare providers, and families is critical for achieving sustainable improvements in oral health outcomes. These findings support the present study’s conclusion that the effectiveness of oral health systems depends not only on the availability of resources but also on the quality and consistency of service delivery processes.

However, the most critical gap identified was the near absence of systematic monitoring and evaluation mechanisms, observed in only a small proportion of CDCs. This deficiency represents a major barrier to continuous quality improvement and accountability within the system. Previous studies have emphasized that monitoring and evaluation are essential components of effective health systems, enabling data-driven decision-making and program refinement [24,25]. Without such mechanisms, even well-implemented interventions cannot be systematically assessed or optimized, limiting their long-term effectiveness. This finding highlights a fundamental weakness in the governance and feedback structures of decentralized oral health systems.

A further interpretation of the coexistence of near-universal preventive coverage with very low monitoring and evaluation (8.15%) and incomplete treatment follow-up (48.89%) is that the system currently operates predominantly within a service-delivery logic rather than a population-health logic. Effort is concentrated on performing discrete, countable activities, such as examinations, fluoride varnish, and supervised tooth brushing, while the feedback functions that would translate these activities into measurable epidemiological gains (surveillance, outcome tracking, recall, and continuity of care) remain underdeveloped. In effect, providers extend the reach of care without closing the loop that would change disease outcomes at the population level. This distinction is supported by evidence from Brazilian primary care, where the provision of dental care for children up to five years of age was positively associated not only with scheduled clinical activity but specifically with population-level planning, programming, and the monitoring and analysis of services [16]. Accordingly, improving oral health outcomes in decentralized child development centres will require reorienting professional practice from output counting toward outcome accountability—embedding routine surveillance, recall, and follow-up as core, funded components of the service model rather than optional administrative tasks.

The analysis of service output factors further underscores the imbalance between preventive coverage and continuity of care. While universal coverage of oral health examinations and fluoride varnish application demonstrates strong adherence to preventive guidelines [23], substantial gaps were identified in referral systems and treatment follow-up. This reflects a broader pattern observed in similar contexts, where high levels of unmet treatment need coexist with limited service utilization [9]. The inability to ensure continuity of care indicates that the system is more effective at detecting and preventing disease than at managing existing conditions, thereby limiting its overall impact on oral health outcomes. The gaps identified in treatment follow-up and continuity of care are also consistent with challenges reported in other decentralized health systems. Studies from Brazil have shown that although preventive dental services can be successfully expanded through primary healthcare networks, ensuring continuity of treatment and timely referral remains a persistent challenge, particularly in socioeconomically disadvantaged and rural communities [15,16]. Similar barriers have been reported in community oral health programs in low- and middle-income countries, where preventive coverage often exceeds access to restorative and follow-up care. These findings suggest that strengthening referral pathways and monitoring systems is a common priority across diverse health system contexts.

From a broader social and environmental perspective, the prevalence of dental caries (57.83%) observed in this study remains high and is consistent with findings from previous studies conducted in Thailand [11,12]. Although the prevalence reported in the present study remains lower than that observed in some disadvantaged populations, international evidence indicates that dental caries continues to represent a major public health challenge among preschool children worldwide. For example, a national survey in Australia reported substantial levels of untreated dental disease among children and highlighted persistent socioeconomic disparities in oral health outcomes despite the availability of universal health services [25]. Similar inequalities have been documented in many countries, suggesting that oral health outcomes are influenced not only by access to services but also by broader social, behavioural, and health system factors.

The multiple regression analysis provides further insight into the determinants of system effectiveness. Service delivery processes (SUMP) emerged as the strongest predictor, followed by home visits by public health and dental personnel (CHS). This finding reinforces the principle that “process quality” is the primary driver of system performance, consistent with health systems theory distinguishing between structural inputs and functional processes [26]. The significance of home visits highlights the importance of extending care beyond institutional settings to the household level, thereby bridging gaps between service provision and health behaviour. This is supported by previous research demonstrating that community outreach and reinforcement by health personnel significantly influence caregiver practices and child health outcomes [24,27].

The strong correlation observed between service delivery processes and home visits suggests a potentially synergistic relationship, where effective in-centre services are complemented by community-based interventions. This finding aligns with integrated care models that emphasize continuity across service settings as a key determinant of health outcomes. It also reflects the broader concept of community participation in health systems, which has been identified as a critical success factor in improving oral health among preschool populations [27].

Several variables input factors, environmental factors, and service accessibility—were not statistically significant predictors. Two considerations apply. First, several of these had near-universal coverage across centres (e.g., examinations and fluoride varnish at 100%), producing limited between-centre variability; with little variation to explain, such variables cannot demonstrate statistical association even where they are theoretically important. This is a property of the sampled system, not evidence of irrelevance. Second, and more substantively, the model explained a modest share of the variance (R^2^ = 0.111), indicating that the eight measured predictors capture only part of what determines effectiveness. This points to the influence of relevant determinants not measured here, such as community health literacy, inter-organizational trust, leadership quality, and local fiscal autonomy, which future models should incorporate. We therefore interpret the regression as identifying two robust correlates of effectiveness (service delivery processes and home visits) rather than as a comprehensive explanatory model. We retain the analysis because the overall model was statistically significant (F(8, 261) = 4.09, *p* < 0.001) and the two significant predictors were stable under multicollinearity diagnostics (VIF < 5.0).

Overall, the findings of this study contribute to the growing body of evidence emphasizing the importance of system-level determinants in shaping health outcomes. While traditional approaches have focused on individual behaviours and clinical indicators, this study demonstrates that organizational processes, governance structures, and community linkages play a critical role in determining system effectiveness. In decentralized settings such as Thailand, where local administrative organizations hold primary responsibility for service delivery, strengthening these system components is essential for achieving sustainable improvements in oral health outcomes among preschool children.

Although this study was conducted in a single province, its principal finding that oral health care system effectiveness is driven primarily by high quality service delivery processes and community based outreach, rather than by the mere availability of resources is consistent with evidence from other decentralized and publicly funded child oral health systems. This suggests that the identified determinants may be applicable to similar decentralized health systems in low- and middle-income settings, where strengthening service delivery and community engagement is likely to improve oral health outcomes among preschool children. In Scotland, the national Childsmile programme, built on universal supervised nursery tooth brushing combined with targeted fluoride varnish and community outreach, has been accompanied by a substantial fall in caries among five-year-old children and by demonstrable cost savings [13], with supervised tooth brushing independently associated with reduced caries experience [14]. The prominence in our model of in-centre service delivery processes—which include daily supervised tooth brushing—and of household-level outreach closely mirrors the active components of that programme. Likewise, Brazil’s decentralized national oral health policy [15], which integrated oral health teams into municipally delivered primary care, illustrates both the gains achievable when oral health is embedded in local service systems and the persistence of access and continuity gaps in rural and disadvantaged areas [27]—a tension that parallels the simultaneous strength in prevention and weakness in follow-up observed here. Interpreted against these international experiences, the determinants identified appear not to be idiosyncratic to Ubon Ratchathani but to reflect generalizable features of how decentralized systems convert inputs into oral health outcomes, suggesting that the principal lever in other settings is likewise the strengthening of process quality and continuity rather than the mere expansion of inputs.

### Limitations

Several limitations should be noted. First, the cross-sectional design precludes causal inference. Second, the 11.1% explained variance indicates that important predictors not included in the model, such as community health literacy, inter-organizational trust, or CDC leadership quality, likely influence system effectiveness. Third, the study was conducted in a single province, and generalizability to other northeastern or national contexts should be approached with caution. Fourth, self-reported data may be subject to social desirability bias. Future research should employ longitudinal or experimental designs, include broader predictor sets, and explore potential mediating pathways between home visits and service delivery processes.

## 5. Conclusions

This study aimed to identify the key determinants influencing the effectiveness of oral health care systems for preschool children in child development centres under decentralized governance in northeastern Thailand. The findings demonstrate that system effectiveness was determined primarily by two factors: the quality of service delivery processes and the integration of proactive community outreach, particularly home visits by public health and dental personnel. While preventive service coverage was universally achieved, critical gaps persisted in monitoring and evaluation, financial sustainability, and continuity of care, which constrained overall system performance. Taken together, these results directly address the study aim by showing that resource availability alone is insufficient to ensure effective system functioning; rather, how services are organized, delivered, and connected to households plays the decisive role in shaping effectiveness. Accordingly, strengthening system effectiveness requires targeted investment in process-quality improvement, the development of robust monitoring and evaluation mechanisms, and the expansion of community-linked service models that bridge institutional care with family-level practices. From a policy perspective, optimizing oral health outcomes in decentralized settings necessitates coordinated action among local administrative organizations, health personnel, and community stakeholders to ensure integrated, accountable, and sustainable service delivery. Future research should build on these findings using longitudinal designs and a broader set of system and behavioural determinants to support evidence-based policy and program development.

## Figures and Tables

**Table 1:** General characteristics of stakeholders involved in the preschool oral health care system in child development centres under local administrative organizations (*n* = 270).

Characteristics	Variables	*n*	%
Sex	- Male	43	15.93
	- Female	227	84.07
Age (years)	- <42	128	47.41
Median = 42, Range = 32, Min = 28, Max = 60	- ≥42	142	52.59
Marital status	- Living alone	92	34.07
	- Living with couple	178	65.93
Educational attainment	- Secondary school and lower	56	20.74
	- Vocational certificate and diploma	92	34.07
	- Bachelor’s degree and above	122	45.19
Occupation	- Merchant employee	164	60.74
	- Agricultural worker	48	17.78
	- Government officer/civil servant	58	21.48
Monthly income (Thai Baht) Median = 10,000, Range = 49,960, Min = 5000; Max = 54,960	- <10,000 THB (about 306 USD)	97	35.93
	- ≥10,000 THB (about 306 USD)	173	64.07
Stakeholder role in relation to the child development centre	- Childcare teacher	30	11.11
	- CDC committee member	60	22.22
	- Village health volunteer (VHV)	60	22.22
	- Dental personnel	28	10.37
	- Public health officer	2	0.74
	- LAO chief administrator	10	3.71
	- Education division director	20	7.41
	- Parent/guardian of preschool child	60	22.22

CDC = Child Development Centre; LAO = Local Administrative Organization; VHV = Village Health Volunteer; THB = Thai Baht.

**Table 2:** Oral health care system factors in child development centres under local administrative organizations (*n* = 270).

Items	Oral Health Care System Factors	Present	
		*n*	%
Input factors of the oral health service system			
1	Designated dental personnel responsible for children’s oral health care	269	99.63
2	Capacity-building training provided for teachers and caregivers	270	100.00
3	Sufficient staffing for oral health service delivery	262	97.04
4	Formally appointed oral health officer within the LAO	244	90.37
5	Budget from the local health security fund for oral health promotion	163	60.37
6	Sufficient budget for procurement of dental health materials	160	59.26
7	Necessary equipment for oral health screening	269	99.63
8	Age-appropriate oral health education materials for preschool children	212	78.52
9	Sufficient oral health promotion supplies for all enrolled children	254	94.07
Oral health service delivery process factors			
10	Clear oral health promotion plans and activities	236	87.41
11	Oral health risk screening system at child enrolment	243	90.00
12	Daily supervised tooth brushing after lunch	270	100.00
13	Referral system for children with oral health problems	216	80.00
14	Coordination with local dental personnel for service planning	255	94.44
15	Parent education and engagement promotion	256	94.81
16	Control system for cariogenic food and beverages	261	96.67
17	Systematic monitoring and evaluation of oral health activities	22	8.15
18	Periodic capacity-building for teaching and care staff	234	86.67
19	Emergency response protocol for acute dental problems during school hours	226	83.70
Oral health service outcome factors			
20	All children receive at least one oral examination per year	270	100.00
21	All at-risk children receive fluoride varnish application	270	100.00
22	Annual oral disease situation report produced	260	96.30
23	Children with identified problems referred for appropriate treatment	159	58.89
24	Continuous treatment and disease prevention follow-up	132	48.89

LAO = Local Administrative Organization.

**Table 3:** Dental Service Accessibility, Agency Support, and Community Resource Factors Supporting Preschool Oral Health Care Systems in Child Development Centres (*n* = 270).

No.	Variable	*n*	%
Dental service access			
Responsible person for accompanying children to dental appointments			
	Childcare teacher	66	24.44
	Parent/caregiver	204	75.56
Mode of transport to dental facility (multiple responses allowed)			
	Bicycle	23	8.52
	Motorcycle	145	53.70
	Private car	53	19.63
	Hired vehicle/Local bus	49	18.15
Agency support			
CDC has previously received oral health promotion supervision from public health/government personnel			
	No	35	12.96
	Yes	235	87.04
CDC has received dental health equipment/material support from relevant agencies (past year)			
	No	16	5.93
	Yes	254	94.07
Types of support received (multiple responses allowed)			
	Oral hygiene supplies (toothbrush, toothpaste, dental floss)	270	100.00
	Dental health educational media (leaflets, posters, videos)	157	58.15
	Training/knowledge-building sessions for parents/caregivers	258	95.56
	Oral health promotion activity organization within CDC	175	64.81
	Oral health examination by dental personnel	260	96.30
	Fluoride varnish application service	261	96.67
	Pit and fissure sealant service	118	43.70
	Budget support from LAO for health activities	139	51.48
	Referral system for children with oral health problems	176	65.19
	Appointment and treatment follow-up system	169	62.59
	Appropriate snack/meal arrangement for dental health within CDC	194	71.85
	Dental health handbooks for parents and teachers	96	35.56
Supporting agencies (multiple responses allowed)			
	Sub-district health promoting hospital	220	81.48
	Community hospital/general hospital	101	37.41
	District public health office	78	28.89
	Provincial public health office	77	28.52
	LAO (municipality/SAO)	163	60.37
	Educational institution (university/college)	90	33.33
	NGO/foundation	12	4.44
Adequacy of dental health equipment/materials received			
	More than sufficient	26	9.63
	Sufficient	144	53.33
	Partially sufficient	49	18.15
	Insufficient	51	18.19
CDC received outreach oral health examination from government health facility in past year			
	No	0	0.00
	Yes	270	100.00

LAO = Local Administrative Organization; SAO = Sub-district Administrative Organization; CDC = Child Development Centre; NGO = Non-governmental Organization.

**Table 4:** Organizational Capacity and System Environment Supporting Preschool Oral Health Care in Child Development Centres (*n* = 270).

No.	CDC Environment Factor	Present	
		*n*	%
1	Written oral health policy or plan	232	85.93
2	Designated primary oral health officer within CDC	236	87.41
3	Oral health risk screening system at enrolment	228	84.44
4	Individual oral health record-keeping system	251	92.96
5	Scheduled annual oral health promotion activity plan	229	84.81
6	Oral health monitoring and follow-up system	244	90.37
7	Referral system for children with oral health problems	257	95.19
8	Coordination system with local dental health agencies	224	82.96
9	Directory of dental personnel and relevant agencies in the area	243	90.00
10	Scheduled outreach visit timetable by dental personnel	220	81.48
11	Training plan for teachers and caregivers on dental health	254	94.07
12	Electronic dental health record management system	148	54.81
13	Automated parental notification system for detected oral health problems	60	22.22
14	Emergency management protocol for acute dental problems during school hours	252	93.33
15	Supervision system for monitoring staff oral health practice	158	58.52
16	Annual budget plan for dental health supplies and equipment	219	81.11
17	Dental health equipment management and adequacy system	236	87.41
18	Regular assessment of children’s oral health knowledge and behaviours	201	74.44
19	Oral health information and guidance provision to parents	262	97.04
20	Regular joint meetings between CDC staff and dental personnel	260	96.30
21	Systematic oral disease surveillance measures	195	72.22
22	Parental participation in the oral health care system	263	97.41
23	Integration of oral health content into teaching curriculum	244	90.37
24	Annual evaluation of oral health care system success	181	67.04

CDC = Child Development Centre. Items classified as: high (≥80%), moderate (60–79%), low (<60%) using Bloom’s criterion-referenced framework.

**Table 5:** Bivariate Correlation Among Oral Health Care System Effectiveness (*n* = 270).

Variable	SUME	SUMI	SUMP	HE	WHS	THS	CHS	FOHS
SUME	1.000							
SUMI	0.368 **	1.000						
SUMP	0.637 **	0.454 **	1.000					
HE	−0.342 **	−0.425 **	−0.476 **	1.000				
WHS	−0.311 **	0.097	−0.226 **	0.367 **	1.000			
THS	0.267 **	−0.116	0.187 **	−0.003	−0.599 **	1.000		
CHS	0.549 **	−0.403 **	0.750 **	0.546 **	0.220 **	−0.126 *	1.000	
FOHS	0.323 **	0.159 **	0.387 **	−0.426 **	−0.143 **	0.135 *	0.650 **	1.000
SUMO	0.129 *	0.186 **	0.258 **	−0.072	0.007	0.001	0.074	0.013

* *p* < 0.05; ** *p* < 0.01. SUME = environmental factors; SUMI = input factors; SUMP = service delivery process; HE = dental health media support; WHS = dental service access; THS = mode of transport; CHS = home visits by public health personnel; FOHS = dental health equipment support; SUMO = oral health care system effectiveness.

**Table 6:** Multiple Linear Regression Coefficients for Factors Associated with Oral Health Care System Effectiveness (*n* = 270).

Predictor Variable	B	SE	β	t	*p*-Value	Collinearity Statistics	
						Tolerance	VIF
Constant	2.021	0.618	—	3.269	0.001 *		
Service delivery process (SUMP)	0.249	0.055	0.458	4.523	<0.001 *	0.332	3.014
Input factors (SUMI)	0.076	0.048	0.119	1.588	0.114	0.610	1.640
Environmental factors (SUME)	−0.010	0.021	−0.036	−0.489	0.625	0.529	1.891
Dental health media support (HE)	0.032	0.076	0.036	0.416	0.678	0.464	2.154
Dental service access (WHS)	0.010	0.165	0.005	0.061	0.952	0.438	2.283
Home visits by public health personnel (CHS)	0.725	0.270	0.303	2.689	0.008 *	0.268	3.726

* *p* < 0.05. SE = standard error; β = standardized coefficient.

## Data Availability

The raw data supporting the conclusions of this article will be made available by the authors on request.

## References

[1] Peres M.A., Macpherson L.M.D., Weyant R.J., Daly B., Venturelli R., Mathur M.R., Listl S., Celeste R.K., Guarnizo-Herreño C.C., Kearns C. (2019). Oral diseases: A global public health challenge. Lancet.

[2] Schroth R.J., Harrison R.L., Moffatt M.E.K. (2009). Oral health of Indigenous children and the influence of early childhood caries on childhood health and well-being. Paediatr. Child Health.

[3] World Health Organization (2022). Global Oral Health Status Report: Towards Universal Health Coverage for Oral Health by 2030.

[4] Zhou Y., Yang J.Y., Lo E.C.M., Lin H.C. (2019). The contribution of life course determinants to early childhood caries: A 2-year cohort study. Caries Res..

[5] Bureau of Dental Health, Department of Health, Ministry of Public Health (2017). Report on the 8th National Oral Health Survey of Thailand.

[6] Bureau of Dental Health, Department of Health, Ministry of Public Health (2022). Guidelines for the Development of Model Early Childhood Development Centres for Oral Health, Volume 1.

[7] Department of Local Administration, Ministry of Interior (2016). Standards for Child Development Centre Operations of Local Administrative Organizations, Fiscal Year 2016.

[8] Khan I.M., Mani S.A., Doss J.G., Danaee M., Kong L.Y.L. (2021). pre-schoolers’ tooth brushing behaviour and association with their oral health: A cross-sectional study. BMC Oral Health.

[9] Kumar V., Ankola A., Sankeshwari R., Jalihal S., Atre S., Mallineni S.K. (2021). Determination of the oral health status and behaviours, treatment needs, and guardians’ perception of oral health among preschool children attending Integrated Child Developmental Scheme Anganwadi centres of Belagavi, South India: A cross-sectional study. J. Clin. Transl. Res..

[10] Kaewthong S., Chimkhong P., Kaewalamul A., Charoensuk D., Mekhwimonkingkaew W. (2022). Factors related to oral health care behaviours among parents of preschool children in child development centres, Warin Chamrap District, Ubon Ratchathani Province. J. Allied Health Sci. Suan Sunandha Rajabhat Univ..

[11] Supak J. (2018). Factors related to oral health status in pre-school children at day care centres, Wichianburi District, Phetchabun Province. Kasalongkham Res. J. Chiangrai Rajabhat Univ..

[12] Ravadee S. (2022). Oral health status and oral health promotion for preschool children in Phra Bu Subdistrict child development centre, Phra Yuen District, Khon Kaen Province. J. Khon Kaen Prov. Health Off..

[13] Anopa Y., McMahon A.D., Conway D.I., Ball G.E., McIntosh E., Macpherson L.M.D. (2015). Improving Child Oral Health: Cost Analysis of a National Nursery Toothbrushing Programme. PLoS ONE.

[14] Ross A.J., Sherriff A., Kidd J., Gnich W., Conway D.I., Macpherson L.M.D. (2023). Evaluating Childsmile, Scotland’s National Oral Health Improvement Programme for Children. Community Dent. Oral. Epidemiol..

[15] Pucca G.A., Gabriel M., de Araujo M.E., de Almeida F.C.S. (2015). Ten Years of a National Oral Health Policy in Brazil: Innovation, Boldness, and Numerous Challenges. J. Dent. Res..

[16] Essvein G., Baumgarten A., Rech R.S., Hilgert J.B., Neves M. (2019). Dental care for early childhood in Brazil: From the public policy to evidence. Rev. Saude Publica.

[17] Cohen J. (1988). Statistical Power Analysis for the Behavioural Sciences.

[18] Checkland P. (1981). Systems Thinking, Systems Practice.

[19] Sawangsuk C. (2002). Theory and Practice in Educational Institution Administration.

[20] Bloom B.S., Hastings J.T., Madaus G. (1971). Handbook on Formative and Summative Evaluation of Student Learning.

[21] Rovinelli R.J., Hambleton R.K. (1977). On the use of content specialists in the assessment of criterion-referenced test item validity. Dutch J. Educ. Res..

[22] Nunnally J.C., Bernstein I.H. (1994). Psychometric Theory.

[23] Cronbach L.J. (1951). Coefficient alpha and the internal structure of tests. Psychometrika.

[24] American Academy of Pediatric Dentistry (2020). Policy on early childhood caries (ECC): Classifications, consequences, and preventive strategies. Pediatr. Dent..

[25] Sonpanao P., Khlewkhern S. (2019). Development of an oral health promotion model for preschool children through community participation in a child development centre under a sub-district administrative organization, Nakhon Ratchasima Province. Thai Dent. Nurse J..

[26] Watt R.G., Daly B., Allison P., Macpherson L.M.D., Venturelli R., Listl S., Weyant R.J., Mathur M.R., Guarnizo-Herreño C.C., Celeste R.K. (2019). Ending the neglect of global oral health: Time for radical action. Lancet.

[27] Wiangdindham W. (2021). Development of an oral health promotion model for preschool children through family and community participation in Kham Pom Subdistrict Child Development Centre, Phra Yuen District, Khon Kaen Province. J. Khon Kaen Prov. Health Off..

